# Lung aeration and ventilation after percutaneous tracheotomy measured by electrical impedance tomography in non-hypoxemic critically ill patients: a prospective observational study

**DOI:** 10.1186/s13613-018-0454-y

**Published:** 2018-11-15

**Authors:** Lars Eichler, Jakob Mueller, Jörn Grensemann, Inez Frerichs, Christian Zöllner, Stefan Kluge

**Affiliations:** 1 Department of Anesthesiology, Center of Anesthesiology and Intensive Care Medicine, University Medical Center Hamburg-Eppendorf, Martinistr. 52, 20251 Hamburg, Germany; 2 Section of Anesthesiology, Tabea Hospital, Hamburg, Germany; 3 Department of Intensive Care Medicine, Center of Anesthesiology and Intensive Care Medicine, University Medical Center Hamburg-Eppendorf, Hamburg, Germany; 4 Department of Anesthesiology and Intensive Care Medicine, University Medical Center Schleswig Holstein, Campus Kiel, Kiel, Germany

## Abstract

**Background:**

Percutaneous dilatational tracheotomy (PDT) may lead to transient impairment of pulmonary function due to suboptimal ventilation, loss of positive end-expiratory pressure (PEEP) and repetitive suction maneuvers during the procedure. Possible changes in regional lung aeration were investigated using electrical impedance tomography (EIT), an increasingly implied instrument for bedside monitoring of pulmonary aeration.

**Methods:**

With local ethics committee approval, after obtaining written informed consent 29 patients scheduled for elective PDT under bronchoscopic control were studied during mechanical ventilation in supine position. Anesthetized patients were monitored with a 16-electrode EIT monitor for 2 min at four time points: (a) before and (b) after initiation of neuromuscular blockade (NMB), (c) after dilatational tracheostomy (PDT) and (d) after a standardized recruitment maneuver (RM) following surgery, respectively. Possible changes in lung aeration were detected by changes in end-expiratory lung impedance (Δ EELI). Global and regional ventilation was characterized by analysis of tidal impedance variation.

**Results:**

While NMB had no detectable effect on EELI, PDT led to significantly reduced EELI in dorsal lung regions as compared to baseline, suggesting reduced regional aeration. This effect could be reversed by a standardized RM. Mean delta EELI from baseline (SE) was: NMB − 47 ± 62; PDT − 490 ± 180; RM − 89 ± 176, values shown as arbitrary units (a.u.). Analysis of regional tidal impedance variation, a robust measure of regional ventilation, did not show significant changes in ventilation distribution.

**Conclusion:**

Though changes of EELI might suggest temporary loss of aeration in dorsal lung regions, PDT does not lead to significant changes in either regional ventilation distribution or oxygenation.

## Introduction

Percutaneous dilatational tracheotomy (PDT) is a standard procedure in critical care patients requiring long-term ventilator support [1, 2]. The intervention is accompanied by repetitive suction maneuvers, application of high oxygen fractions, reduced ventilation and loss of positive end-expiratory pressure (PEEP) [3]. These procedural factors might cause formation of atelectasis, possibly leading to impaired respiratory function immediately thereafter [4].

Electrical impedance tomography (EIT) detects areas of reduced lung aeration by measuring a parallel reduction in electrical resistance, which reflects the fact that electrically conducting structures are less stretched and more densely arranged [5, 6]. The level of end-expiratory lung impedance (EELI) was shown to correlate with the intrathoracic gas volume at this time point, i.e., the end-expiratory lung volume (EELV) [7]. Impedance changes between inspiration and expiration (tidal variation) are a measure of global and regional tidal volumes [8]. EIT is therefore used in real-time imaging of impaired aeration in diseased lungs of, e.g., patients with acute respiratory distress syndrome (ARDS) [9, 10], helps in prompt evaluation of therapeutic interventions such as recruitment maneuvers [11, 12] and PEEP titration [13] and may give additional information for decision-making when considering extracorporeal circulation [14].

Because long-term ventilated critically ill patients are prone to pulmonary complications, and especially to formation of atelectases [15], we aimed to assess the effects of PDT on lung aeration. We prospectively investigated changes in global and regional lung aeration during a standardized tracheotomy procedure using EIT. Since the procedure is generally well tolerated, we hypothesized that EIT does not show changes in local ventilation due to tracheotomy in the studied patients (Table 1).

**Table 1:** Patient characteristics

	BMI (kg/ m^2^)	Age (years)	SAPS II	TISS-28	Horovitz index (mm Hg)	Compliance (ml/mbar)	Duration of MV (h)
Mean	26	62	43	17	296	35	272
SD	± 6	± 11	± 13	± 6	± 97	± 8	± 131

*BMI* body mass index; *SAPS II* simplified acute physiology score II [36]; *TISS-28* therapeutic intervention scoring system [37]; *Horovitz index* ratio of arterial partial pressure of oxygen and inspiratory fraction of oxygen; *SD* standard deviation; *MV* mechanical ventilation

## Materials and methods

This prospective observational study was approved by the local ethics committee of the Hamburg Chamber of Physicians, Germany, and registered on ClinicalTrials.gov (NCT02161328). Written informed consent was obtained from patients’ legal representatives.

Mechanically ventilated patients undergoing PDT with the Ciaglia single-step dilator technique (Ciaglia Blue Rhino^®^ G2, Cook Medical, Bloomington, IN, USA) were enrolled. Tracheal cannulation and insertion of the dilator during PDT were visually guided by bronchoscopy (Olympus BF-P60, Olympus Medical Systems Corporation, Tokyo, Japan). Patients with hemodynamic instability, significant pulmonary morbidity defined as COPD GOLD 2 or greater, asthma bronchiale or known interstitial lung disease and metallic foreign bodies such as implantable electronic cardiac devices and sternal wires were excluded. A 16-electrode EIT monitoring belt (PulmoVista 500™ EIT belt, Drägerwerk, Lübeck, Germany) was attached at a level above the intermammillary line and connected to a portable EIT monitor (PulmoVista 500™, Drägerwerk, Lübeck, Germany). The size of the EIT belt was chosen according to the manufacturer’s recommendations.

Anesthesia for PDT was maintained with sufentanil and propofol. Neuromuscular blockade was induced with rocuronium at a dose of 1 mg/kg ideal body weight (IBW). Before data acquisition, patients were placed in supine position with a shoulder pad to ensure adequate cervical reclination and to avoid positional changes in between measurements. Mechanical ventilation was set to a pressure controlled mode (BIPAP, Evita V500, Drägerwerk, Lübeck, Germany) with a standardized PEEP level of 8 cm H_2_O and an inspiratory pressure aiming for tidal volumes of 6 ml/kg IBW. A pressure support level was not used. After endotracheal suction to clear secretions, a standardized recruitment maneuver consisting of a 30-s phase of three sighs (airway pressure: 30/15 cm H_2_O; respiratory rate: 6 min^−1^; and inspiration expiration ratio: 1:2) was performed. This maneuver was repeated after PDT.

Using the standard ventilator settings described above, EIT measurements were obtained for 2 min at 30 Hz at four predefined time points as follows:

(a) before neuromuscular blockade (baseline), (b) 2 min. after neuromuscular blockade (NMB), (c) after PDT (PDT) and (d) after a final standardized recruitment maneuver following PDT (RM).

We recorded changes in EELI in arbitrary units (a.u.) from baseline as well as the tidal variation in impedance given also in a.u. in all image pixels. To analyze regional effects of the procedure, EIT images were primarily divided into a ventral and a dorsal region of interest (ROI) and compared to baseline measurements. Additionally, ventrodorsal distribution of changes in EELI and tidal impedance variation were calculated within all 32 rows of the scan volume. Furthermore, the distribution of ventilation within the thoracic cross section was characterized by the center of ventilation (CoV, given in % of anteroposterior chest diameter) [16]. Values above 50% indicate a dorsally distributed ventilation and values below 50% ventral distribution of ventilation. The detailed description of all EIT measures calculated is given in [17].

Blood gas analysis (BGA) samples were obtained at each of the four time points, and the Horovitz indices as the ratio of partial pressure of oxygen (paO_2_) divided by the inspiratory fraction of oxygen (FiO_2_) as a measure of oxygenation were calculated.

Data analysis was performed using GraphPad Prism 6.0 (GraphPad Software, La Jolla, CA, USA). Data were tested for normal distribution with the Kolmogorov–Smirnov normality test. Data are presented as means ± standard deviation (SD) or median and interquartile range. Data were tested using *t* tests or *U* tests, as appropriate. For changes of parameters between the time points, repeated measures analyses of variance (ANOVA) were used. Two-tailed *p* values < 0.05 were considered as statistically significant. An a priori power calculation (G*Power 3.1.9.2, Universities of Kiel and Dusseldorf, Germany) revealed a sample size of 29 to be sufficient to detect a difference in EELI of 30 a.u. between the time points for an estimated standard deviation of 80 a.u. for a repeated measures, within factors ANOVA with an error probability for *α* = 0.05 and 1 − *β* = 0.80.

## Results

**Fig. 1:**
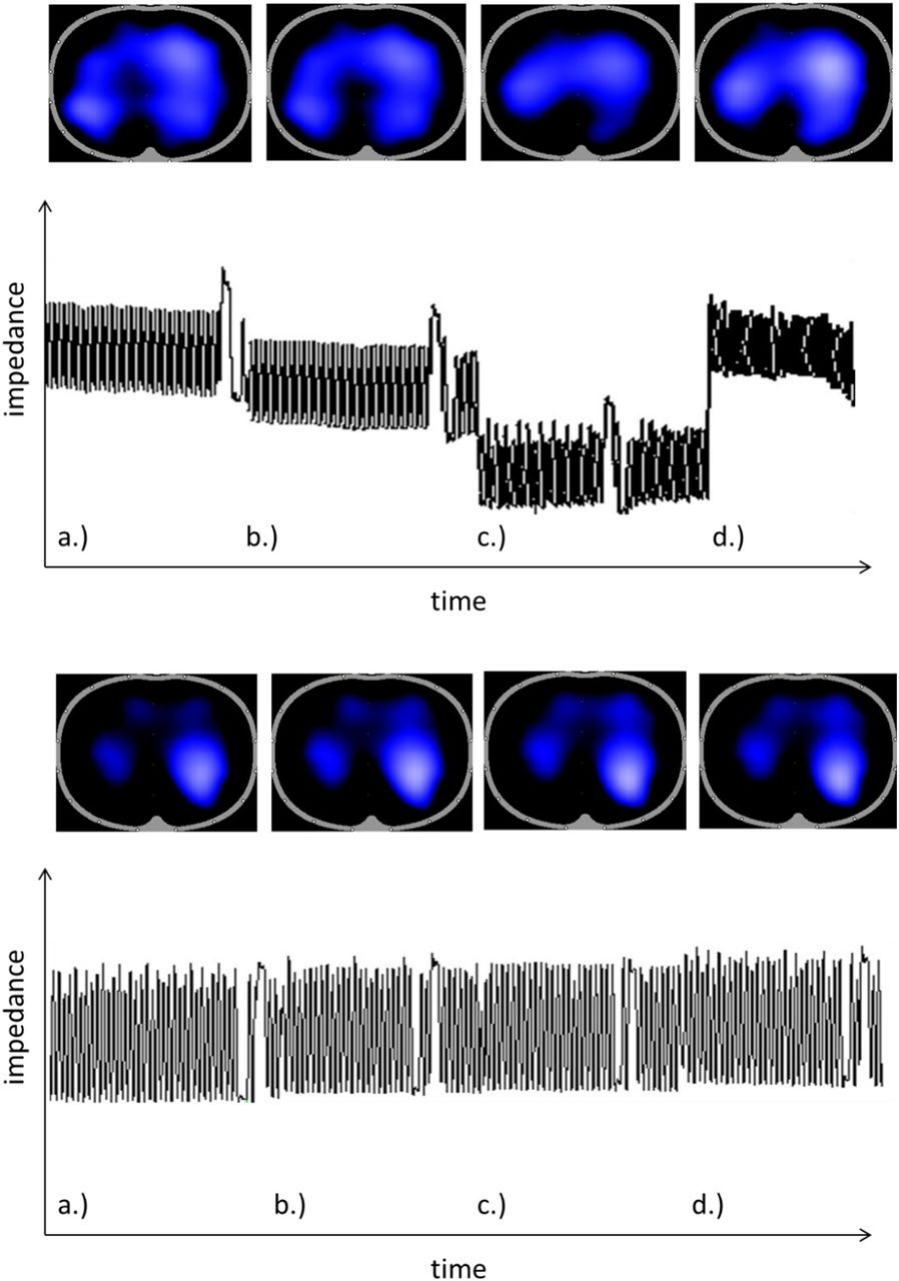
Exemplary sequences of four original impedance tracings at the four study time points acquired in two patients. **a** baseline: before application of muscle relaxant, **b** NMB: 2 min after application of muscle relaxant, **c** PDT: after tracheotomy and **d** RM: after a final standardized recruitment maneuver. The corresponding graphical displays of regional ventilation (“minute images”) are shown above. The top patient (age 57 years, BMI 24 kg/m^2^; duration of MV 234 h) showed a pronounced decrease in global EELI after PDT which was reversed by application of a RM. The bottom patient (age 54 years; BMI 19 kg/m^2^, duration of MV 228 h) did not show any marked changes in EELI among the four study time points. This latter result was detected in the majority of the studied patients and represents the overall study findings

A total of 30 consecutive patients were included in this study. One patient was excluded due to injury of the membranous part of the trachea which was diagnosed upon bronchoscopic inspection immediately after the insertion of the tracheal cannula. Figure 1 exemplarily displays sequences of four original waveforms showing tidal variations in global impedance in two of the examined patients. The corresponding minute EIT images of tidal impedance variations within the thoracic cross section are shown at the top of each panel. In the example patient shown in the top panel, a marked decrease in EELI is seen in the third examination phase right after PDT. This reduction in aerated lung tissue was reversed by the applied RM as shown in the last part of the waveform. The other example patient shown in the bottom panel does not show any pronounced changes in EELI between the four time points.

**Fig. 2:**
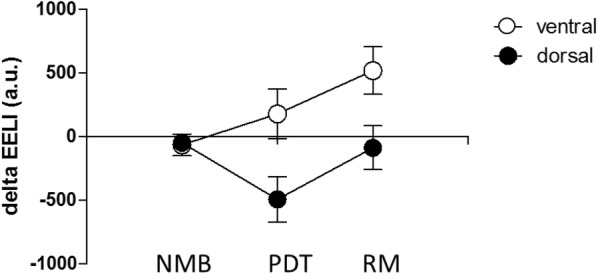
Changes in end-expiratory lung impedance (delta EELI). Data are given in arbitrary units (a.u.) presented as mean (error bars indicate standard error of the mean) compared to baseline measurements (NMB) after neuromuscular blockade, (PDT) after tracheotomy and (RM) after a subsequent recruitment maneuver, *n* = 29. Mean changes were significantly different from baseline values in dorsal lung parts after PDT (*p* = 0.01) and in ventral parts after the recruitment maneuver (*p* = 0.01), RM ANOVA

Global EELI changes did not significantly differ from baseline values (NMB: − 116.7 ± 120.8; PDT: − 338.9 ± 341.7; RM: 406.7 ± 319.9; *p* > 0.05). To analyze regional effects of the procedure, EIT images of all 29 patients were divided into a ventral and a dorsal region of interest (ROI) and compared to baseline measurements. While neuromuscular blockade had no effect on regional end-expiratory impedance levels (mean changes in EELI (a.u.) from baseline (SD): dorsal − 47.5 (61.7); ventral − 63.7 (86.4)), PDT led to a significant decrease in impedance within the dorsal aspect of the measured thoracic cross section, while a slight nonsignificant increase within the ventral ROI was observed (dorsal − 492.6 (179.1), *p* = 0.01; ventral 179.8 (200), *p* = 0.38). EIT measurements following the applied RM also revealed regional effects of this procedure: The observed loss in dorsal lung impedance could be restored to baseline levels (− 47.5 (61.1) *p* = 0.62) while ventral lung regions showed a significant increase in volume above baseline levels (519.4 (190) *p* = 0.01) (Fig. 2).

**Fig. 3:**
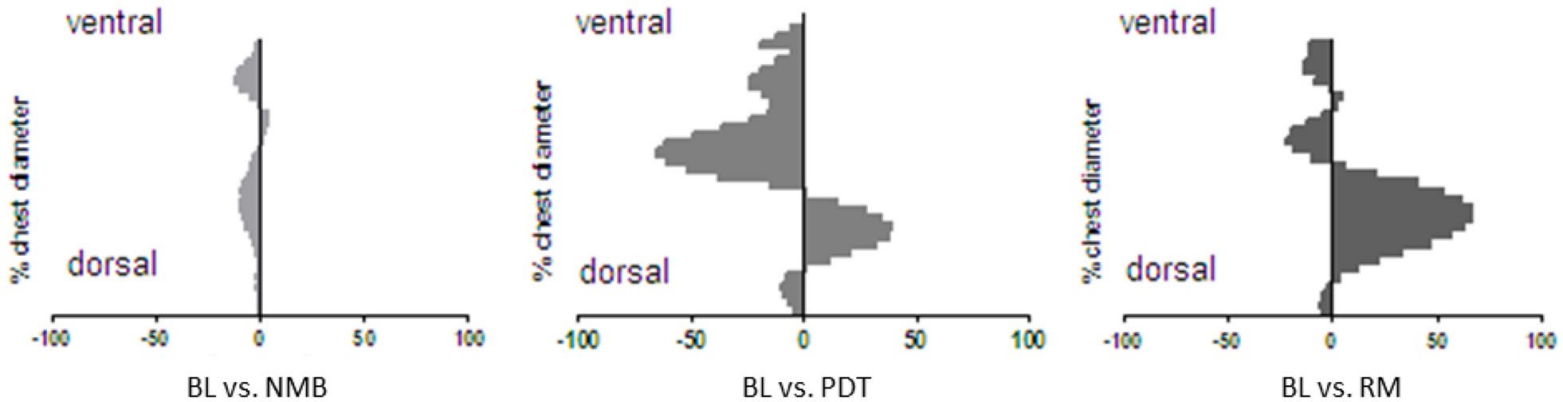
Mean regional changes in EELI compared to baseline measurements (NMB) after neuromuscular blockade, (PDT) after tracheotomy and (RM) after a subsequent recruitment maneuver, *n* = 29

This regionally dissimilar effect of PDT and subsequent RM was visible with greater detail when displaying the changes in EELI within each of the 32 horizontal rows of the EIT image (Fig. 3). While NMB led to insignificant changes of EELI as compared to baseline, reduction in EELI by PDT and its reversal upon application of the standardized RM were visible within dorsal rows of the scan. Parallel increases in EELI within ventral rows of the scans suggest a dorsoventral redistribution of aeration by PDT. While dorsal reductions in aerated lung tissue are reversed by RM, ventral aeration seems augmented even further above baseline levels.

**Fig. 4:**
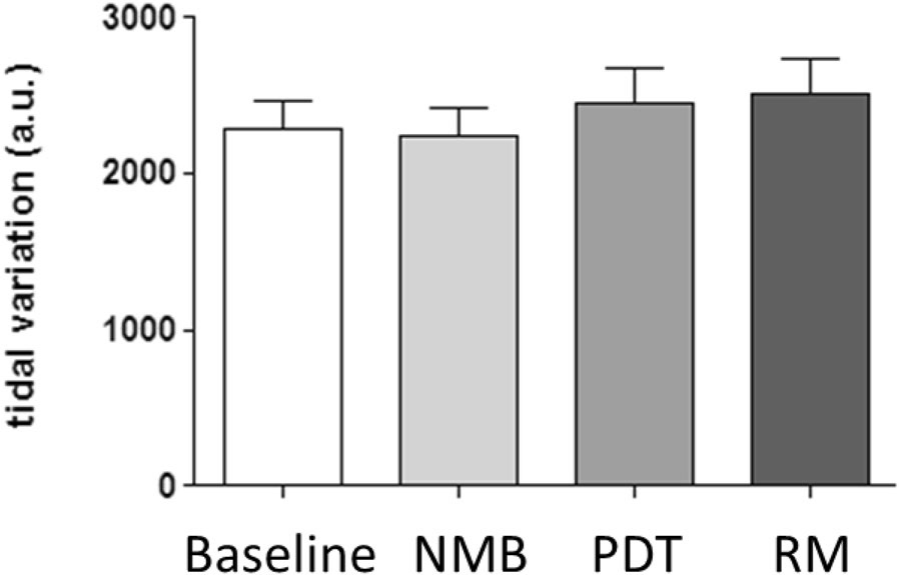
Mean global tidal variation during each measurement phase: **a** before neuromuscular blockade (baseline), **b** after neuromuscular blockade (NMB), **c** after tracheotomy (PDT) and **d** after a subsequent recruitment maneuver (RM) (*n* = 29; RM ANOVA; *p* > 0.05)

**Fig. 5:**
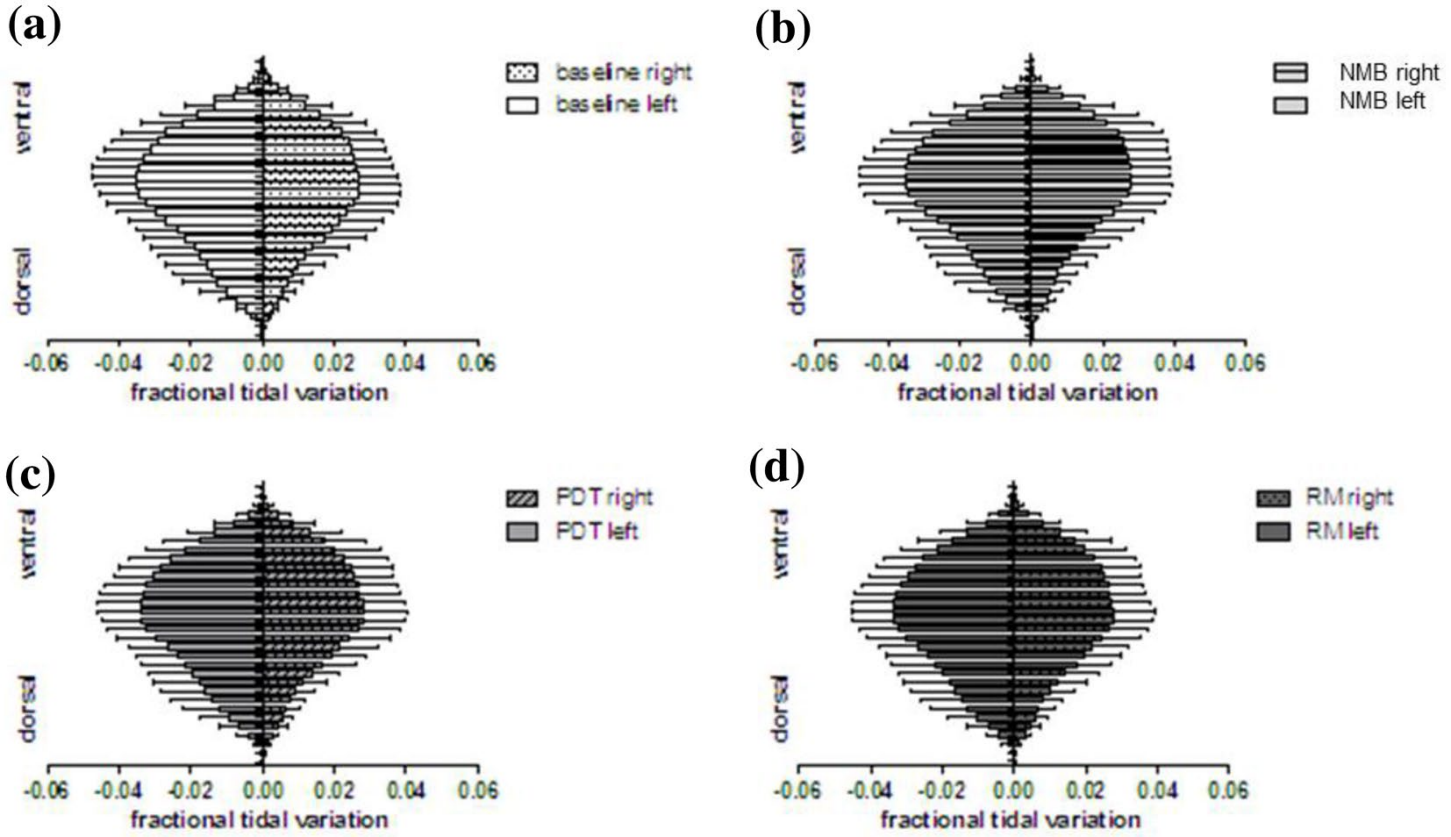
Regional tidal variation within 32 horizontal rows of the right and left halves of EIT image in each measurement phase **a** before neuromuscular blockade (baseline), **b** after neuromuscular blockade (NMB), **c** after tracheotomy (PDT) and **d** after a subsequent recruitment maneuver (RM). No significant changes in regional tidal variation were detected among the four time points of our standard PDT procedure (*n* = 29; RM ANOVA; *p* > 0.05)

Analysis of global and regional tidal variation in thoracic impedance was performed to test whether changes in EELI also affected regional ventilation distribution. In contrast to EELI, tidal variation in impedance did not exhibit significant changes in global nor regional ventilation compared with baseline in either of the three phases of the procedure (Figs. 4, 5).

**Fig. 6:**
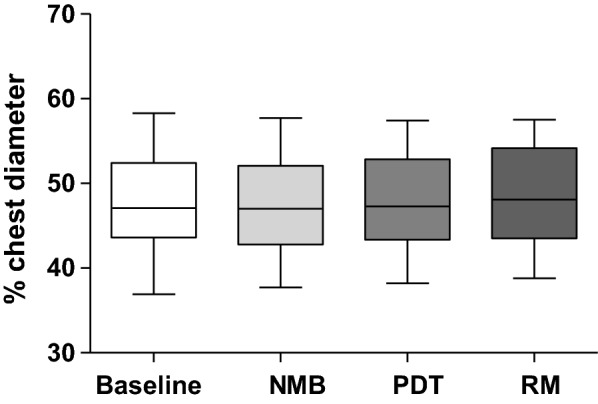
Center of ventilation (CoV) describes the distribution of ventilation within the scanned thoracic cross section. A value below 50% indicates a ventrally distributed ventilation, while values above 50% indicate a preferential distribution of ventilation to the dorsal aspects of the scanned volume. No significant changes in CoV were detected among the four time points of our standard PDT procedure (*n* = 29; *p* > 0.05; RM ANOVA)

Accordingly, the geometrical center of tidal distribution within the thoracic cross section, the center of ventilation (CoV), did not show significant differences among the four time points (Fig. 6).

**Fig. 7:**
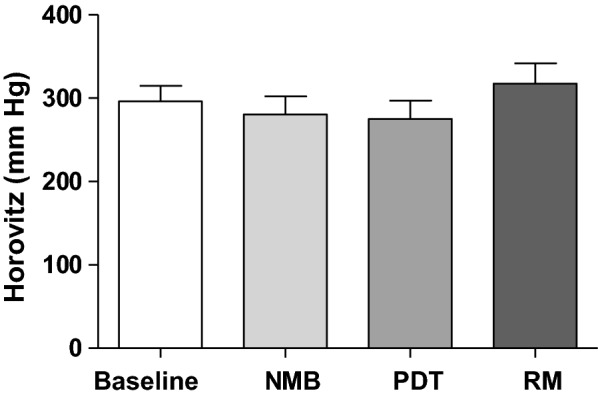
Horovitz index (paO_2_/F_i_O_2_) at each measurement phase: (baseline) before neuromuscular blockade, (NMB) after neuromuscular blockade, (PDT) after tracheotomy and (RM) after a subsequent recruitment maneuver, *n* = 29 (*p* > 0.05; repeated measures ANOVA)

Comparison of BGA measurements at the four distinct time points of the procedure did not show significant changes in the Horovitz indices (Fig. 7).

## Discussion

This study showed that PDT under bronchoscopic guidance led to regionally dissimilar alterations of EELI, while tidal variation in ventilation and oxygenation remained unaffected. The observed reduction in EELI in dependent lung areas is suggestive of collapse caused by repetitive suction maneuvers and loss of PEEP [8, 18]. While effects of changes in positioning of the patients or even just the arms on the EELI are described [19], we thoroughly avoided any alterations of patients’ positioning in between the four measurements. Since bronchoscopy and concomitant suction maneuvers did not focus on dorsal lung areas, and macroaspiration and hemorrhage did not occur, the observed changes in EELI distribution most likely result from the same gravitational factors that cause formation of dorsal atelectases in supine, mechanically ventilated patients [20]. A parallel increase in EELI within the ventral aspect of the scan volume indicates increased aeration. The immediate application of a standardized RM was able to reverse dorsal reduction in EELI. Since pleural pressures imposed on ventral lung aspects are significantly lower, in these regions elevation of airway pressure during RM resulted in higher transpulmonary pressures and further increase in EELI [21].

Interestingly, analysis of tidal variation in impedance did not indicate significant changes in regional ventilation during any of the three distinct phases of the procedure. This is in contrast to previous studies which showed formation of dorsally located atelectasis upon induction of anesthesia and neuromuscular blockade in supine subjects [22–24]. The widely accepted concept is that paralysis of the diaphragm with subsequent compression of dorsobasal lung areas by upper abdominal organs and a decrease in thoracic diameter are the most important pathophysiological causes of “anesthesia-induced atelectasis” [25, 26]. A possible explanation for our differing observations may be given from the patient cohort studied: Patients in our study had been on respirator support with mostly controlled modes of ventilation for a mean time of more than 11 days, and general anesthesia was induced well before application of a neuromuscular blocking agent. Mandatory ventilation itself leads to diaphragmatic dysfunction [27] while general anesthesia, even without the use of muscle relaxants, causes a decrease in functional residual capacity and the formation of atelectasis [28]. Therefore, one can reason that diaphragm function was already altered by prolonged ventilator therapy and transition to mandatory ventilation upon induction of anesthesia for PDT, so that the net effect of adding a non-depolarizing muscle relaxant was too small to become apparent during our study. Another explanation for the missing effect of neuromuscular blockade could be the timing of our measurement. Though a maximum effect of rocuronium may be assumed 2 min after injection of the dosage we used, redistribution of ventilation upon the resulting changes in thoracic geometry may have taken longer than this [29]. Therefore, we may have missed the delayed effect of muscular paralysis upon lung aeration. Moreover, spatial resolution of EIT may not be sufficient to detect minor changes as it has been shown to be inferior to computed tomography scans, which are considered as gold standard of lung imaging. This means formation of subtle atelectases may have been missed in our investigation.

An explanation for a missing impact of PDT itself on regional lung ventilation (though pathophysiological reasoning—as explained above—would make marked changes in lung aeration very likely) is hard to give: High oxygen fractions are known to promote atelectasis formation during preoxygenation for intubation [30], and this effect can be minimized by a reduction of the FiO_2_ [31]. Since we did perform PDT under a FiO_2_ of 1.0, one would expect to detect formation of resorption atelectases. However, these might take longer to evolve to full extent as was shown in patients, who developed gradually progressive atelectases during the course of 45 min after exposure to a FiO_2_ of 0.8 [32].

Atelectases develop if the lung’s closing capacity (CC) drops below the functional residual capacity (FRC). FRC can be increased by increasing PEEP while CC depends on various factors, i.e., on pulmonary disease, age, or patients’ positioning. To allow for inter-individual comparison, we ventilated all patients with a standardized PEEP during the intervention. The PEEP level chosen for our study may have been high enough to keep FRC well above the CC in our patients, therefore preventing the formation of atelectases even with a FiO_2_ of 1.0. While a fall in EELI may just reflect aeration loss, regional tidal volume changes (and tidal impedance variation) need not change if FRC is maintained and the lung units still operate in the linear part of regional pressure–volume curves.

It has been shown that a RM before PDT may improve patients’ gas exchange [33], presumably due to a resolution of atelectases. Interestingly, atelectases and hypoxia during PDT are seldom reported. Atelectases are believed to occur in approximately 2% when using bronchoscopic guidance [34] and hypoxia in approximately 1%. Regarding intra-procedural mortality, hypoxia does not play a role [35]. Furthermore, it has been shown for hypoxemic patients that tracheotomy even improves oxygenation with an increase in the Horovitz index of approximately 35 mmHg [36].

Our findings of unchanged lung ventilation may explain the rare occurrence of hypoxia with consecutive hypoxemia during PDT. Therefore, should hypoxemia occur during the procedure, and other factors, i.e., intratracheal hemorrhage or even technical malfunctions, should be considered.

Our study has certain limitations. The oxygenation of the studied patients was in a stable condition, and it would be of interest how lung aeration changed with changes in oxygenation during PDT. All patients were ventilated with a fixed PEEP value. We did not measure functional residual capacity and did not determine a “best PEEP” which plays a role concerning atelectasis formation and therefore a decrease in lung aeration. We excluded patients with a preexisting pulmonary pathology. However, these patients presumably may benefit from visualization of lung aeration by EIT by the application of a targeted ventilatory approach. Since atelectases and hypoxia are rare intra-procedural complications of PDT, our sample size may not have been sufficient.

## Conclusion

The PDT procedure did not result in detectable changes in regional lung ventilation. Oxygenation indices remained adequate during the intervention. Therefore, despite ventilation with a FiO_2_ of 1.0 and repetitive suctioning during the procedure, no detectable atelectases and hypoxia occurred. This supports the notion that bedside PDT is a well-tolerated intervention in critically ill patients with low risks of atelectases and hypoxia. EIT during PDT should be evaluated further in high-risk patient groups, i.e., patients with a preexisting pulmonary morbidity.

## Abbreviations

a.u.: arbitrary units
BGA: blood gas analysis
Δ: changes in (delta)
EELI: end-expiratory lung impedance
EIT: electrical impedance tomography
NB: neuromuscular blockade
PBW: predicted body weight
PEEP: positive end-expiratory pressure
PDT: percutaneous dilatational tracheotomy
RM: recruitment maneuver
ROI: region of interest
SD: standard deviation of the mean
SE: standard error of the mean

## References

[1] Putensen C (2014). Percutaneous and surgical tracheostomy in critically ill adult patients: a meta-analysis. Crit Care.

[2] Braune S, Kluge S (2012). Update on tracheotomy. Med Klin Intensivmed Notfmed.

[3] Durbin CG (2005). Techniques for performing tracheostomy. Respir Care.

[4] Ferraro F (2004). Assessment of ventilation during the performance of elective endoscopic-guided percutaneous tracheostomy: clinical evaluation of a new method. Chest.

[5] Hinz J (2003). End-expiratory lung impedance change enables bedside monitoring of end-expiratory lung volume change. Intensive Care Med.

[6] Bodenstein M, David M, Markstaller K (2009). Principles of electrical impedance tomography and its clinical application. Crit Care Med.

[7] Grivans C (2011). Positive end-expiratory pressure-induced changes in end-expiratory lung volume measured by spirometry and electric impedance tomography. Acta Anaesthesiol Scand.

[8] Bikker IG (2011). Electrical impedance tomography measured at two thoracic levels can visualize the ventilation distribution changes at the bedside during a decremental positive end-expiratory lung pressure trial. Crit Care.

[9] Pulletz S (2012). Dynamics of regional lung aeration determined by electrical impedance tomography in patients with acute respiratory distress syndrome. Multidiscip Respir Med.

[10] Bellani G, Mauri T, Pesenti A (2012). Imaging in acute lung injury and acute respiratory distress syndrome. Curr Opin Crit Care.

[11] Cinnella G (2015). Physiological effects of the open lung approach in patients with early, mild, diffuse acute respiratory distress syndrome: an electrical impedance tomography study. Anesthesiology.

[12] Dargaville PA, Rimensberger PC, Frerichs I (2010). Regional tidal ventilation and compliance during a stepwise vital capacity manoeuvre. Intensive Care Med.

[13] Zhao Z (2014). The EIT-based global inhomogeneity index is highly correlated with regional lung opening in patients with acute respiratory distress syndrome. BMC Res Notes.

[14] Franchineau G (2017). Bedside contribution of electrical impedance tomography to setting positive end-expiratory pressure for extracorporeal membrane oxygenation-treated patients with severe acute respiratory distress syndrome. Am J Respir Crit Care Med.

[15] Woodring JH, Reed JC (1996). Types and mechanisms of pulmonary atelectasis. J Thorac Imaging.

[16] Frerichs I (2006). Lung volume recruitment after surfactant administration modifies spatial distribution of ventilation. Am J Respir Crit Care Med.

[17] Frerichs I (2017). Chest electrical impedance tomography examination, data analysis, terminology, clinical use and recommendations: consensus statement of the TRanslational EIT developmeNt stuDy group. Thorax.

[18] Corley A (2014). Lung volume changes during cleaning of closed endotracheal suction catheters: a randomized crossover study using electrical impedance tomography. Respir Care.

[19] Vogt B (2016). Influence of torso and arm positions on chest examinations by electrical impedance tomography. Physiol Meas.

[20] Klingstedt C (1990). The influence of body position and differential ventilation on lung dimensions and atelectasis formation in anaesthetized man. Acta Anaesthesiol Scand.

[21] Gattinoni L (1995). Effects of positive end-expiratory pressure on regional distribution of tidal volume and recruitment in adult respiratory distress syndrome. Am J Respir Crit Care Med.

[22] Lundquist H (1995). CT-assessment of dependent lung densities in man during general anaesthesia. Acta Radiol.

[23] Brismar B (1985). Pulmonary densities during anesthesia with muscular relaxation—a proposal of atelectasis. Anesthesiology.

[24] Tokics L (1987). Lung collapse and gas exchange during general anesthesia: effects of spontaneous breathing, muscle paralysis, and positive end-expiratory pressure. Anesthesiology.

[25] Bendixen HH, Hedley-Whyte J, Laver MB (1963). Impaired oxygenation in surgical patients during general anesthesia with controlled ventilation. a concept of atelectasis. N Engl J Med.

[26] Hedenstierna G, Edmark L (2016). Effects of anesthesia on the respiratory system. Best Pract Res Clin Anaesthesiol.

[27] Vassilakopoulos T, Petrof BJ (2004). Ventilator-induced diaphragmatic dysfunction. Am J Respir Crit Care Med.

[28] Lindberg P (1992). Atelectasis and lung function in the postoperative period. Acta Anaesthesiol Scand.

[29] Caruana L (2015). The time taken for the regional distribution of ventilation to stabilise: an investigation using electrical impedance tomography. Anaesth Intensive Care.

[30] Rothen HU (1998). Airway closure, atelectasis and gas exchange during general anaesthesia. Br J Anaesth.

[31] Edmark L (2003). Optimal oxygen concentration during induction of general anesthesia. Anesthesiology.

[32] Franchi F (2012). Recruitment maneuver in prevention of hypoxia during percutaneous dilational tracheostomy: randomized trial. Respir Care.

[33] Gobatto AL (2016). Ultrasound-guided percutaneous dilational tracheostomy versus bronchoscopy-guided percutaneous dilational tracheostomy in critically ill patients (TRACHUS): a randomized noninferiority controlled trial. Intensive Care Med.

[34] Simon M (2013). Death after percutaneous dilatational tracheostomy: a systematic review and analysis of risk factors. Crit Care.

[35] Bellani G (2013). Effect of percutaneous tracheostomy on gas exchange in hypoxemic and non-hypoxemic mechanically ventilated patients. Respir Care.

[36] Le Gall JR, Lemeshow S, Saulnier F (1993). A new simplified acute physiology score (SAPS II) based on a European/North American multicenter study. JAMA.

[37] Miranda DR, de Rijk A, Schaufeli W (1996). Simplified therapeutic intervention scoring system: the TISS-28 items—results from a multicenter study. Crit Care Med.

